# Supplementary material for: An Agenda for Research of Uncovered Epidemiological Patterns of Tick-Borne Pathogens Affecting Human Health
Source: Int J Environ Res Public Health. 2023 Jan 26;20(3):2206. doi: 10.3390/ijerph20032206 (PMC9915995; doi:10.3390/ijerph20032206)
Supplement: Supplementary file 1 [file ijerph-20-02206-s001.zip › ijerph-2103735-supplementary.pdf]

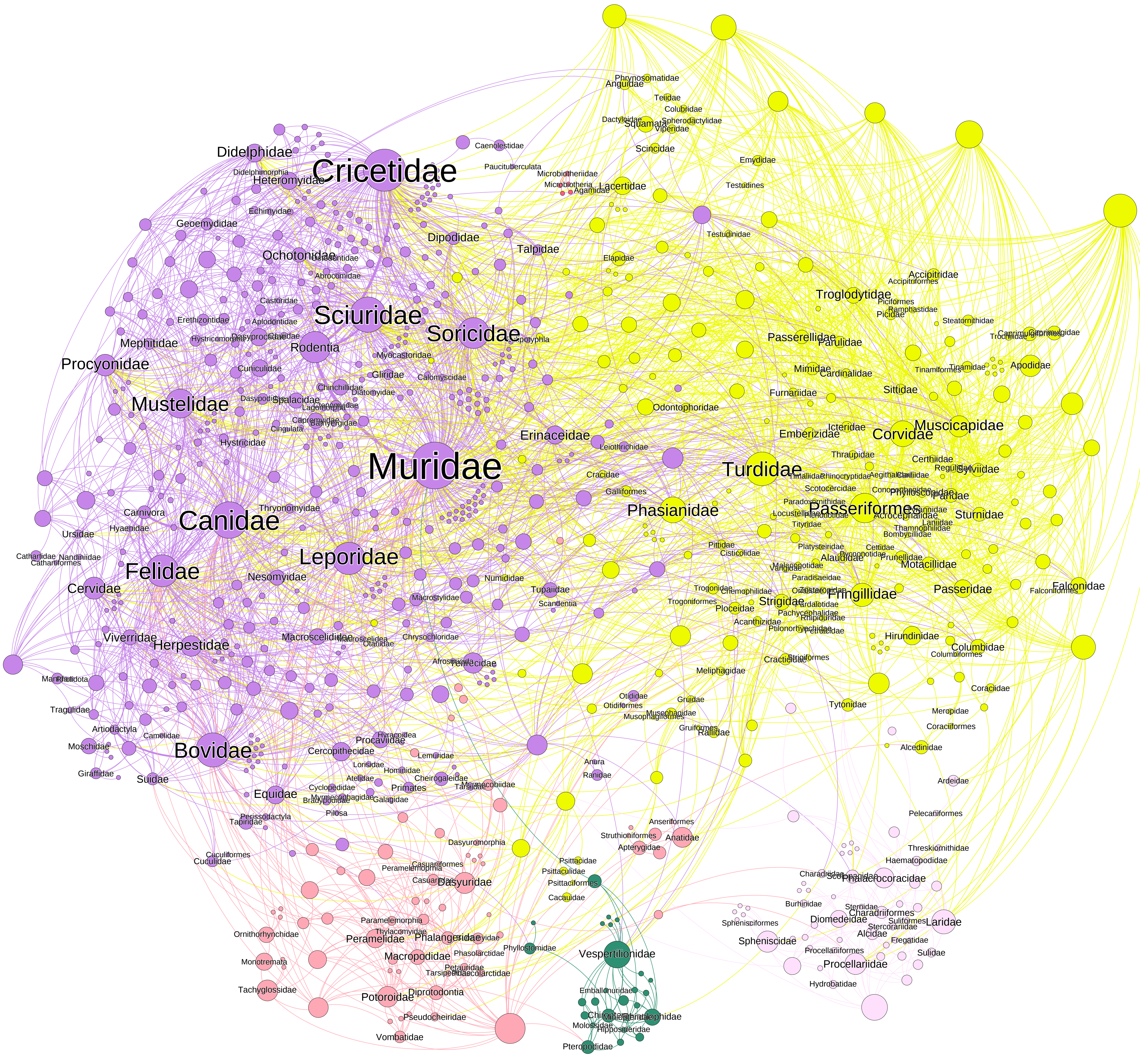

Phrynosomatidae
Anguidae
Teiidae
Colubridae
Dactyloidae
Squamata
Spherodactylidae
Viperidae
Scincidae
Emydidae
Caenolestidae
Paucituberculata
Microbiotheriidae
Microbiotheria
Agamidae
Lacertidae
Testudines
Testudinidae
Didelphidae
Didelphimorphia
Cricetidae
Heteromyidae
Echimyidae
Geoemydidae
Dipodidae
Talpidae
Elapidae
Ochotonidae
Abrocomidae
Accipitridae
Accipitriformes
Troglodytidae
Piciformes
Ramphastidae
Picidae
Steatornithidae
Trochilidae
Castoridae
Erethizontidae
Aplodontiidae
Sciuridae
Soricidae
Mephitidae
Hystricomorpha
Dasyproctidae
Rodentia
Eulipotyphla
Passerellidae
Parulidae
Procyonidae
Cuniculidae
Myocastoridae
Gliridae
Calomyscidae
Mimidae
Cardinalidae
Tinamiformes
Tinamidae
Apodidae
Chinchillidae
Diatomyidae
Furnariidae
Sittidae
Mustelidae
Spalacidae
Lagomorpha
Odontophoridae
Capromyidae
Cingulata
Icteridae
Emberizidae
Corvidae
Muscicapidae
Hystricidae
Erinaceidae
Leiothrichidae
Thraupidae
Certhiidae
Regulidae
Vireonidae
Muridae
Turdidae
Cracidae
Galliformes
Scotocercidae
Phylloscopidae
Paridae
Paradoxornithidae
Thryonomyidae
Carnivora
Hyaenidae
Phasianidae
Passeriformes
Locustellidae
Acrocephalidae
Sturnidae
Canidae
Tityridae
Laniidae
Thamnophilidae
Bombycillidae
Ursidae
Catharidae
Cathartiformes
Nandiniidae
Pittidae
Cisticolidae
Platysteiridae
Cettidae
Leporidae
Alaudidae
Prunellidae
Motacillidae
Felidae
Nesomyidae
Numididae
Cervidae
Tupaiidae
Scandentia
Trogonidae
Trogoniformes
Ploceidae
Strigidae
Fringillidae
Passeridae
Falconidae
Falconiformes
Macrostylidae
Acanthizidae
Pachycephalidae
Rhipiduridae
Viverridae
Herpestidae
Macroscelididae
Macroscelidea
Chrysochloridae
Hirundinidae
Columbidae
Columbiformes
Tenrecidae
Afrosoricida
Cracticidae
Strigiformes
Meliphagidae
Manidae
Pholidota
Coraciidae
Otididae
Otidiformes
Gruidae
Tytonidae
Musophagidae
Musophagiformes
Meropidae
Tragulidae
Gruiformes
Rallidae
Coraciiformes
Artiodactyla
Camelidae
Hyracoidea
Procaviidae
Moschidae
Bovidae
Cercopithecidae
Lemuridae
Alcedinidae
Lorisidae
Hominidae
Anura
Ranidae
Giraffidae
Suidae
Atelidae
Cheirogaleidae
Myrmecobiidae
Tarsiidae
Cyclopedidae
Primates
Ardeidae
Equidae
Bradypodidae
Galagidae
Pilosa
Perissodactyla
Tapiridae
Pelecaniformes
Anseriformes
Cuculiformes
Cuculidae
Dasyuromorphia
Struthioniformes
Apterygidae
Anatidae
Threskiornithidae
Haematopodidae
Casuariiformes
Peramelemorphia
Casuariidae
Dasyuridae
Psittacidae
Psittaculidae
Psittaciformes
Cacatuidae
Charadriidae
Phalacrocoracidae
Burhinidae
Sternidae
Charadriiformes
Paramelemorphia
Thylacomyidae
Ornithorhynchidae
Peramelidae
Phalangeridae
Spheniscidae
Diomedeidae
Suliformes
Stercorariidae
Laridae
Alcidae
Phyllostomidae
Vespertilionidae
Spheniscidae
Fregatidae
Macropodidae
Phascolarctidae
Procellariiformes
Procellariidae
Sulidae
Monotremata
Petauridae
Tarsipedidae
Hydrobatidae
Tachyglossidae
Emballonuridae
Potoroidae
Diprotodontia
Rhinolophidae
Pseudocheiridae
Molossidae
Hipposideridae
Vombatidae
Pteropodidae

Figure S1. The network of the reported hosts of every species of the tick genus Ixodes in the world. Nodes (circles) are either species of ticks or families of vertebrates, and their size is proportional to their importance in the network; the labels of the tick species have been removed to improve the figure's clarity. Lines (links) represent the use of a host by a tick. The colours correspond to clusters, groups of tick-vertebrates that tend to appear together more frequently than with others; this is a property of the network known as "modularity".
